# TPD52 as a Therapeutic Target Identified by Machine Learning Shapes the Immune Microenvironment in Breast Cancer

**DOI:** 10.1111/jcmm.70333

**Published:** 2025-01-05

**Authors:** Jie Xia, Xudong Zhou

**Affiliations:** ^1^ Department of Respiratory and Critical Care Medicine, National Clinical Research Center of Respiratory Disease Key Laboratory of Pulmonary Diseases of Health Ministry, Tongji Hospital, Tongji Medical College, Huazhong University of Science and Technology Wuhan China; ^2^ Department of Thyroid and Breast Surgery Tongji Hospital, Tongji Medical College, Huazhong University of Science and Technology Wuhan China

**Keywords:** breast cancer, immune microenvironment, machine learning, therapeutic target, TPD52

## Abstract

Breast cancer (BRCA) is one of the most common malignancies and a leading cause of cancer‐related mortality among women globally. Despite advances in diagnosis and treatment, the heterogeneity of BRCA presents significant challenges for effective management and prognosis. Recent studies emphasise the critical role of the tumour microenvironment, particularly immune cells, in influencing tumour behaviour and patient outcomes. This study uses machine learning–based methodologies to investigate the role of tumour protein D52 (TPD52) as a pivotal immune regulator in BRCA. We employed single‐cell RNA sequencing (scRNA‐seq) to characterise the immune landscape of breast tumours and identify differentially expressed genes (DEGs) associated with TPD52. Our findings indicate that TPD52 may modulate immune cell infiltration and the tumour immune landscape, impacting tumour aggression and patient survival. Furthermore, we performed in vitro validation to elucidate the functional implications of TPD52. By integrating computational analysis with experimental validation, this research highlights TPD52's potential as a biomarker for therapeutic intervention and provides insights into its role in immune regulation within the BRCA microenvironment.

## 1 Introduction

Breast cancer (BRCA) remains one of the most prevalent malignancies and a leading cause of cancer‐related mortality among women worldwide. Despite advances in early detection and treatment, the heterogeneity of BRCA poses significant challenges to effective management and prognosis [1]. Recent studies have highlighted the importance of the tumour microenvironment (TME), particularly the roles of immune cells, in influencing tumour behaviour and patient outcomes [2].

The TME is a pivotal factor in breast cancer treatment, significantly influencing tumour progression, metastasis and response to therapies. Comprising a complex network of immune cells, fibroblasts, endothelial cells and extracellular matrix components, the TME can create conditions that either support or hinder tumour growth [2]. In breast cancer, the TME is often characterised by a heterogeneous population of immune cells, which can contribute to either tumour‐promoting inflammation or immune suppression. This interplay can impact the effectiveness of treatments such as chemotherapy, targeted therapies and immunotherapies [3]. Among these interactions, immune regulation has emerged as a critical area of investigation, with specific immune checkpoints and regulatory molecules being identified as potential therapeutic targets [4].

In this context, TPD52 (tumour protein D52) has garnered attention as a putative immune regulator in BRCA. Preliminary findings suggest that TPD52 may influence immune cell infiltration and modulate the tumour immune landscape, thereby impacting tumour aggression and patient survival [5]. However, its precise role and the underlying mechanisms still need to be better understood.

This study aims to elucidate the role of TPD52 as an immune regulator in BRCA using advanced machine learning–based methodologies. We will employ single‐cell RNA sequencing (scRNA‐seq) to characterise the immune landscape of breast tumours and identify differentially expressed genes (DEGs) associated with TPD52. Furthermore, we will investigate the prognostic significance of TPD52 in various datasets and explore its functional implications through in vitro validation studies. By integrating computational analysis with experimental validation, this research seeks to contribute to understanding TPD52's role in BRCA and its potential as a biomarker for therapeutic intervention.

## 2 Results

### 2.1 Establishment of the IE Patterns

**FIGURE 1:**
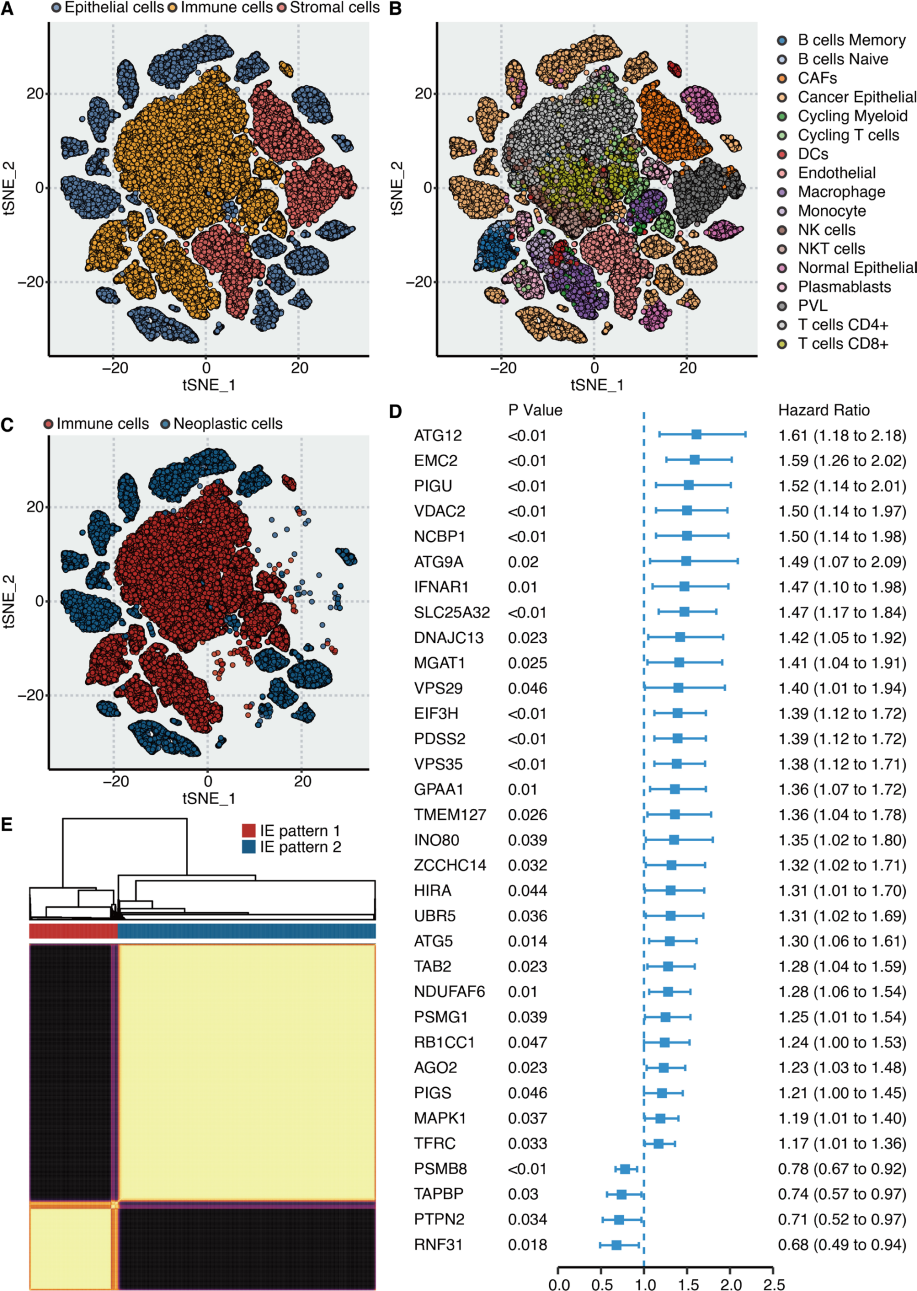
Establishment of the IE patterns. (A) t‐SNE plot shows the distribution of epithelial cells, immune cells and stromal cells by scRNA‐seq analysis. (B) t‐SNE plot shows the distribution of microenvironment cells by scRNA‐seq analysis. (C) t‐SNE plot shows the distribution of immune and neoplastic cells by scRNA‐seq analysis. (D) Univariate Cox regression analysis on IE genes. (E) Clustering results of IE patterns by k‐means method.

The distribution of epithelial cells, immune cells and stromal cells in BRCA is revealed by scRNA‐seq analysis (Figure 1A). Seventeen microenvironment cells are defined in BRCA by scRNA‐seq analysis (Figure 1B). The distribution of immune and neoplastic cells in BRCA is revealed by scRNA‐seq analysis in Figure 1C,D. Univariate Cox regression analysis on IE genes determined that 29 genes were significantly hazardous genes while four genes were significantly favourable genes (Figure 1D). Clustering results of IE patterns by the k‐means method are shown in Figure 1E.

### 2.2 Identification of TPD52 as a Hazardous Marker

**FIGURE 2:**
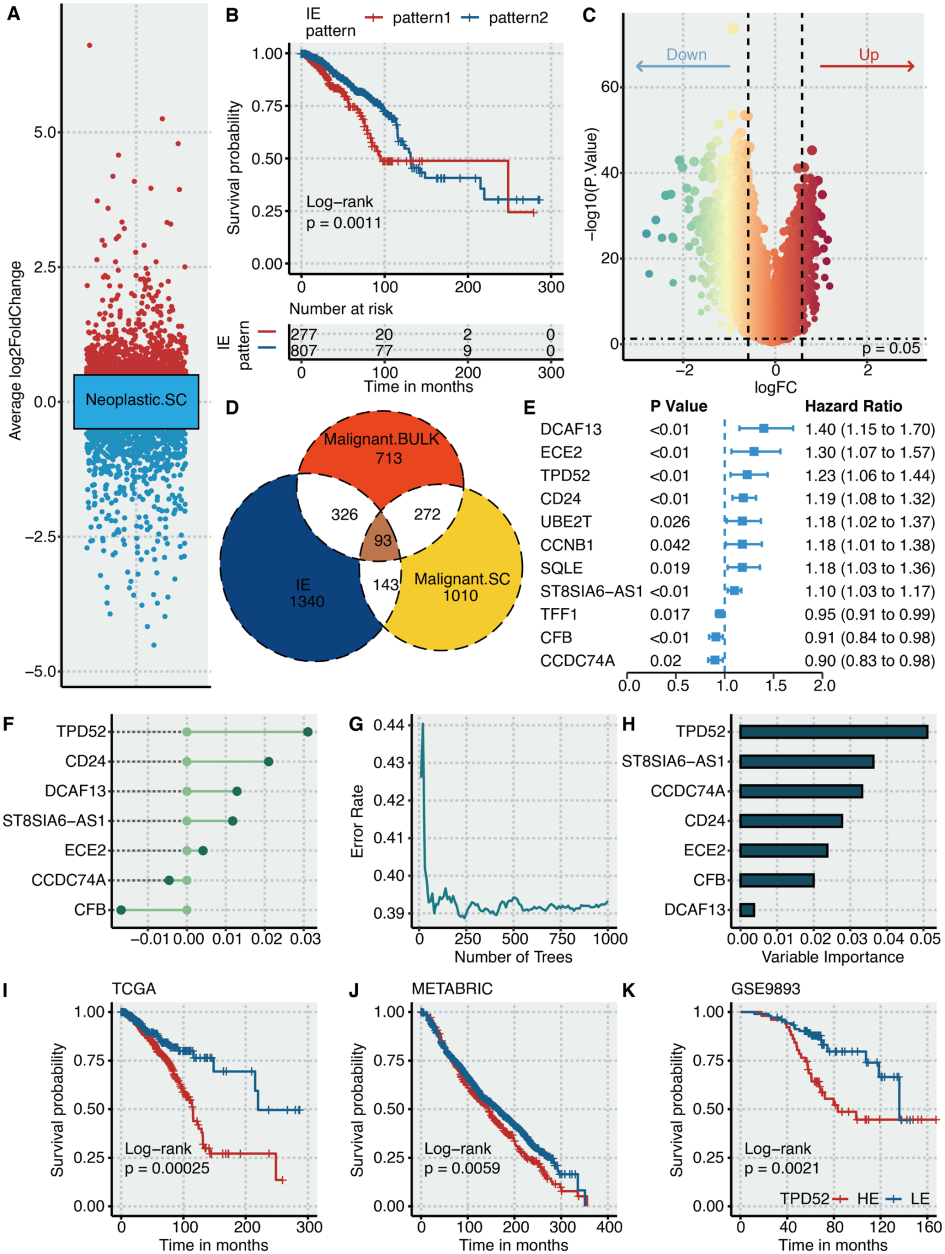
Identification of TPD52 as a hazardous marker. (A) Volcano plot shows the DEGs between immune and neoplastic cells by scRNA‐seq analysis. (B) Survival curves of IE patterns. (C) Volcano plot shows the DEGs between IE patterns. (D) Venn plot shows the intersected genes among IE patterns‐related DEGs, scRNA‐seq‐related DEGs and bulk‐seq‐related DEGs. (E) Univariate Cox regression analysis on intersected genes. (F) CoxBoost analysis on prognostic intersected genes. (G) Error rate and number of trees in random survival forest analysis. (H) Random survival forest analysis on prognostic intersected genes. (I) Survival curves of TPD52‐based groups in TCGA. (J) Survival curves of TPD52‐based groups in METABRIC. (K) Survival curves of TPD52‐based groups in GSE9893.

Volcano plot shows the DEGs between immune and neoplastic cells by scRNA‐seq analysis (Figure 2A). BRCA patients in IE pattern 1 had significantly shortened survival time than in IE pattern 2 (Figure 2B). Volcano plot shows the DEGs between IE patterns (Figure 2C). Venn plot shows 93 intersected genes among IE patterns‐related DEGs, scRNA‐seq‐related DEGs and bulk‐seq‐related DEGs (Figure 2D). Univariate Cox regression analysis on intersected genes determined that eight genes were significantly hazardous genes while three genes were significantly favourable genes (Figure 2E). CoxBoost analysis was performed on the dimension reduction of prognostic intersected genes and came to the seven most potent genes (Figure 2F). Error rate and number of trees in RSF analysis are shown in Figure 2G. RSF analysis was performed on the dimension reduction of the prognostic intersected genes and came to the seven most potent genes (Figure 2H). In both methods, TPD52 was identified as the most potent gene and was, therefore, selected for further exploration. BRCA patients with high TPD52 expression had significantly shortened survival time in TCGA (Figure 2I), METABRIC (Figure 2J) and GSE9893 datasets (Figure 2K).

### 2.3 Functional Annotation of TPD52

**FIGURE 3:**
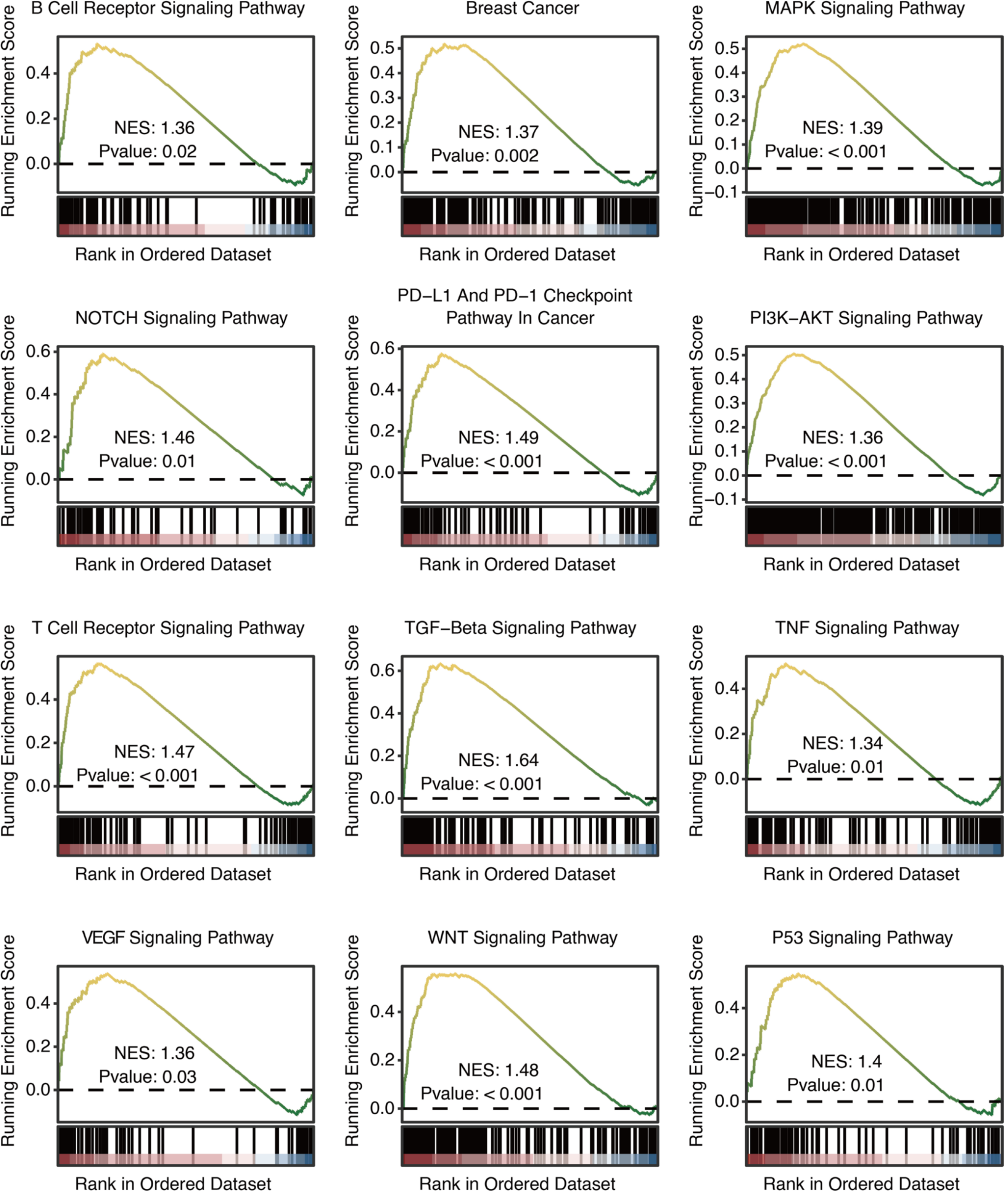
Functional annotation of TPD52. GSEA of oncogenic and immunogenic pathways related to TPD52.

GSEA of oncogenic and immunogenic pathways related to TPD52 revealed that B cell receptor signalling pathway, breast cancer, MAPK signalling pathway, NOTCH signalling pathway, PD‐L1 and PD‐1 checkpoint pathway in cancer, PI3K‐Akt signalling pathway, T‐cell receptor signalling pathway, TGF‐beta signalling pathway, TNF signalling pathway, VEGF signalling pathway, WNT signalling pathway and p53 signalling pathway were highly connected to TPD52 (Figure 3). The estimated AUC for chemotherapy drugs, including Sapitinib, LCL161, Lapatinib, Acetalax and Temozolomide, was notably lower in high SLFN12‐expressed patients (Figure S1).

### 2.4 Mutation Characteristics of TPD52

**FIGURE 4:**
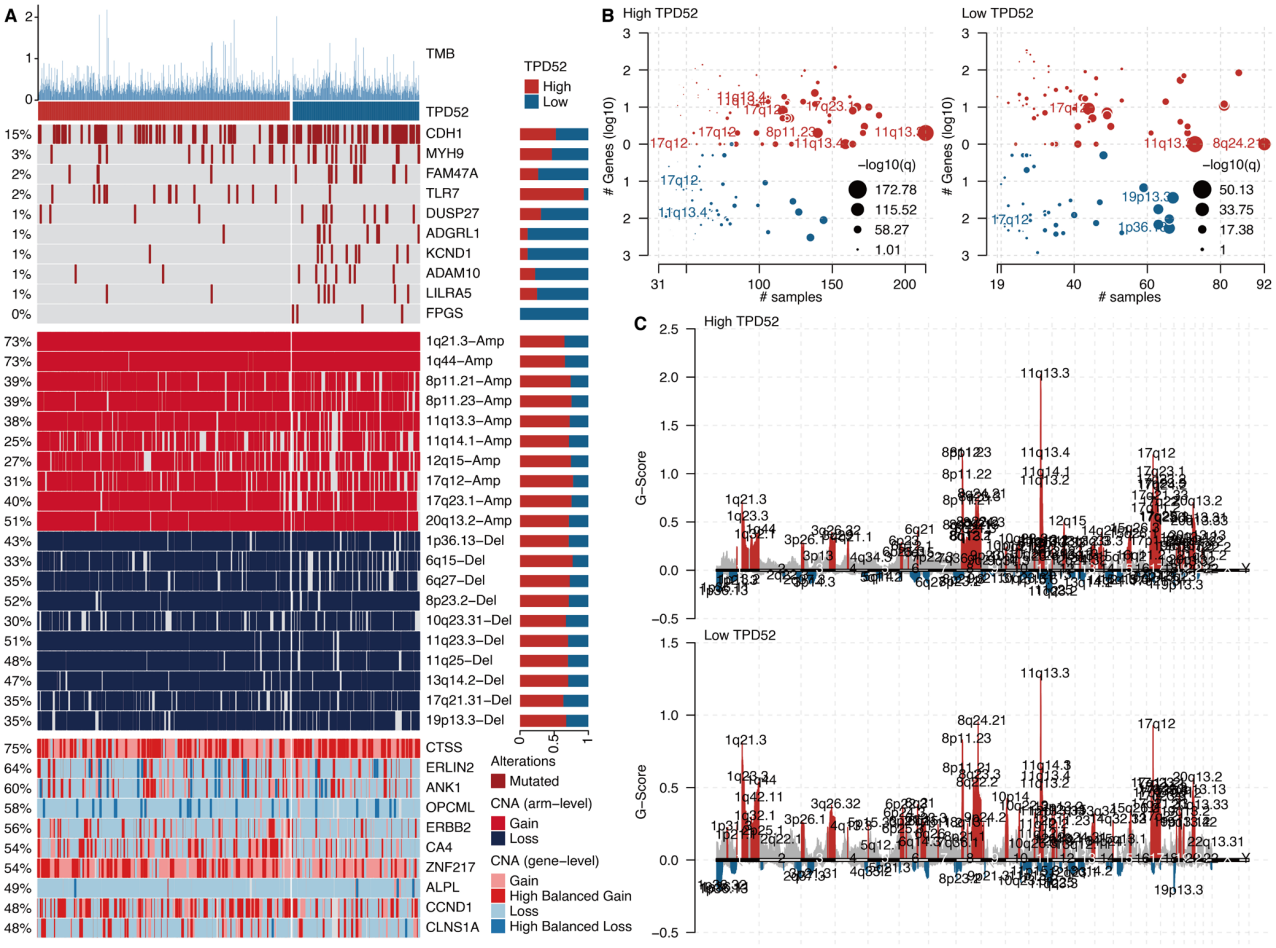
Mutation characteristics of TPD52. (A) Waterfall plot shows the overall mutation patterns in TPD52‐based groups. (B) Bubble plot shows the mutation sites in TPD52‐based groups. (C) G‐score of the mutation sites in TPD52‐based groups.

Waterfall plot shows the overall mutation patterns in TPD52‐based groups, in which CDH1 had the highest mutation rate (15%) in BRCA patients with high TPD52 expression (Figure 4A). The bubble plot shows the mutation sites in TPD52‐based groups, in which 1q21.3 and 1q44 were highly amplified in BRCA patients with high TPD52 expression (Figure 4B). G‐score of the mutation sites in TPD52‐based groups is shown in Figure 4C.

### 2.5 Immune Characteristics of TPD52

**FIGURE 5:**
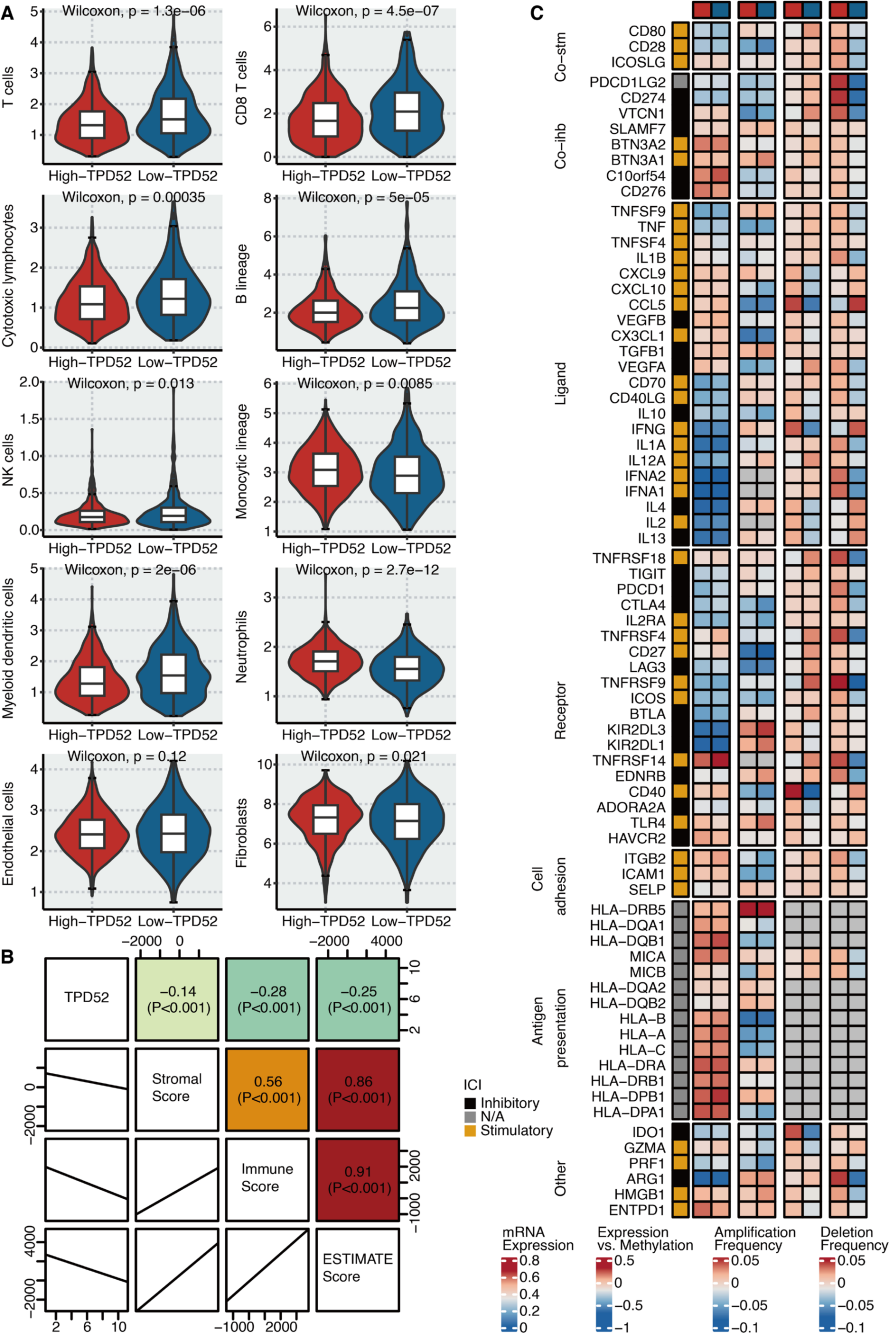
Immune characteristics of TPD52. (A) Violin plot shows the immune cell abundance in TPD52‐based groups. (B) The correlation between TPD52 and immune score, stromal score and ESTIMATE score. (C) Heatmap shows the relationship between TPD52 and immune modulators.

In our study, we focused on several key immune cell types—T cells, cytotoxic lymphocytes, B cells, NK cells, monocytes, neutrophils and myeloid dendritic cells—when assessing immune infiltration scores in the context of BRCA. The selection of these immune cell types for assessing immune infiltration scores in breast cancer is grounded in their fundamental roles in immune responses and their implications for tumour behaviour and patient outcomes [6]. By analysing the presence and activity of these cells within the TME, we can gain insights into the immune landscape of breast cancer, which is vital for understanding tumour progression, response to therapy and potential therapeutic strategies. The abundance of T cells, cytotoxic lymphocytes, B cells, NK cells, monocytes, neutrophils and myeloid dendritic cells was significantly lower in BRCA patients with high TPD52 expression (Figure 5A). There is a significant negative correlation between TPD52 and immune score, stromal score and ESTIMATE score (Figure 5B). TPD52 was significantly positively associated with immune modulators (Figure 5C).

### 2.6 In Vitro Validation on TPD52

**FIGURE 6:**
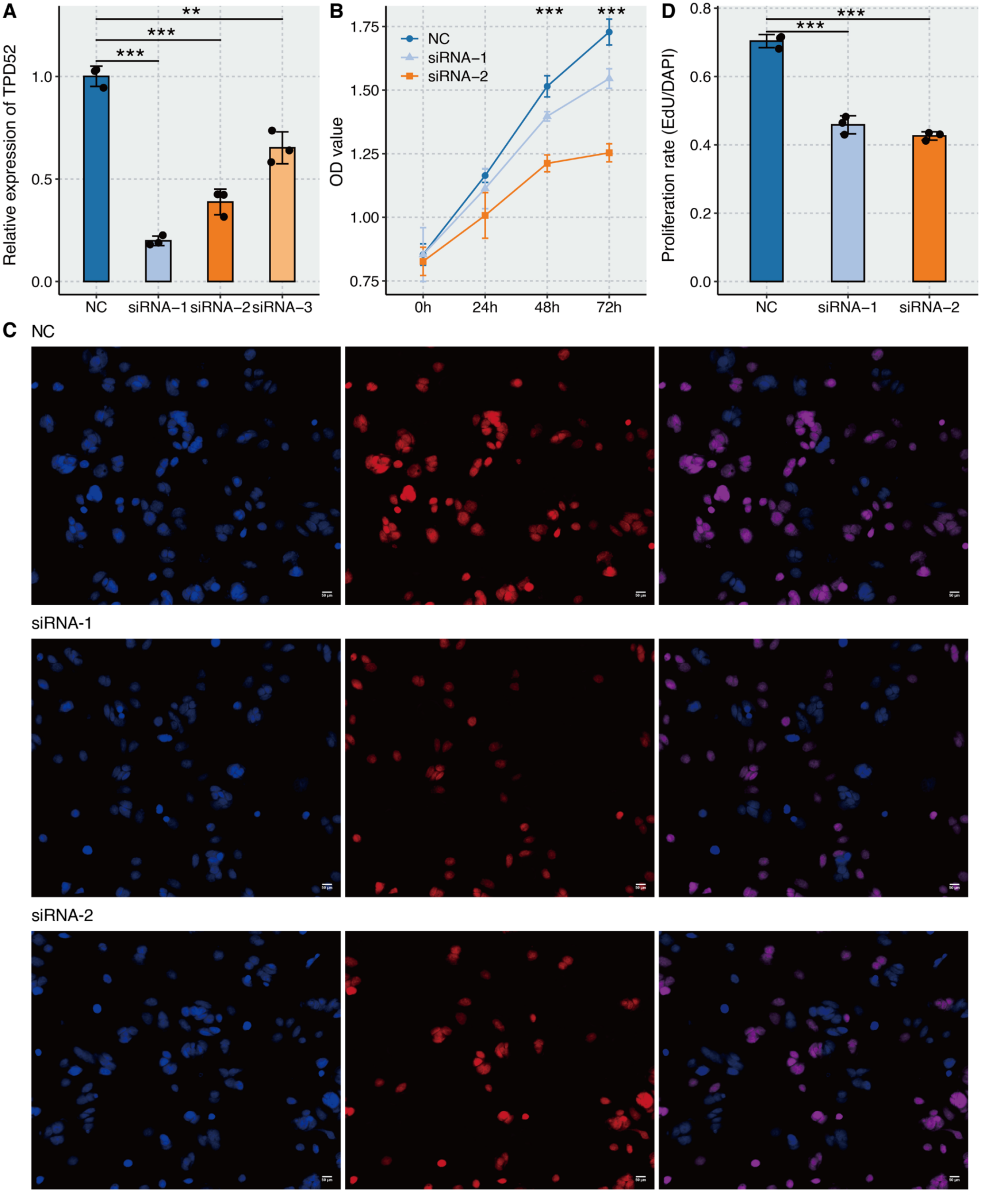
In vitro validation on TPD52. (A) RT‐qPCR assay reveals the relative RNA expression of TPD52 in NC and three siRNA groups. (B) OD value of MCF‐7 cells in NC and two siRNA groups by CCK‐8 assay. (C) EdU assay reveals the proliferated MCF‐7 cells in NC and two siRNA groups. Blue represents Hoechst, red represents EdU and purple represents merged. (D) Statistical analysis of EdU assay.

**FIGURE 7:**
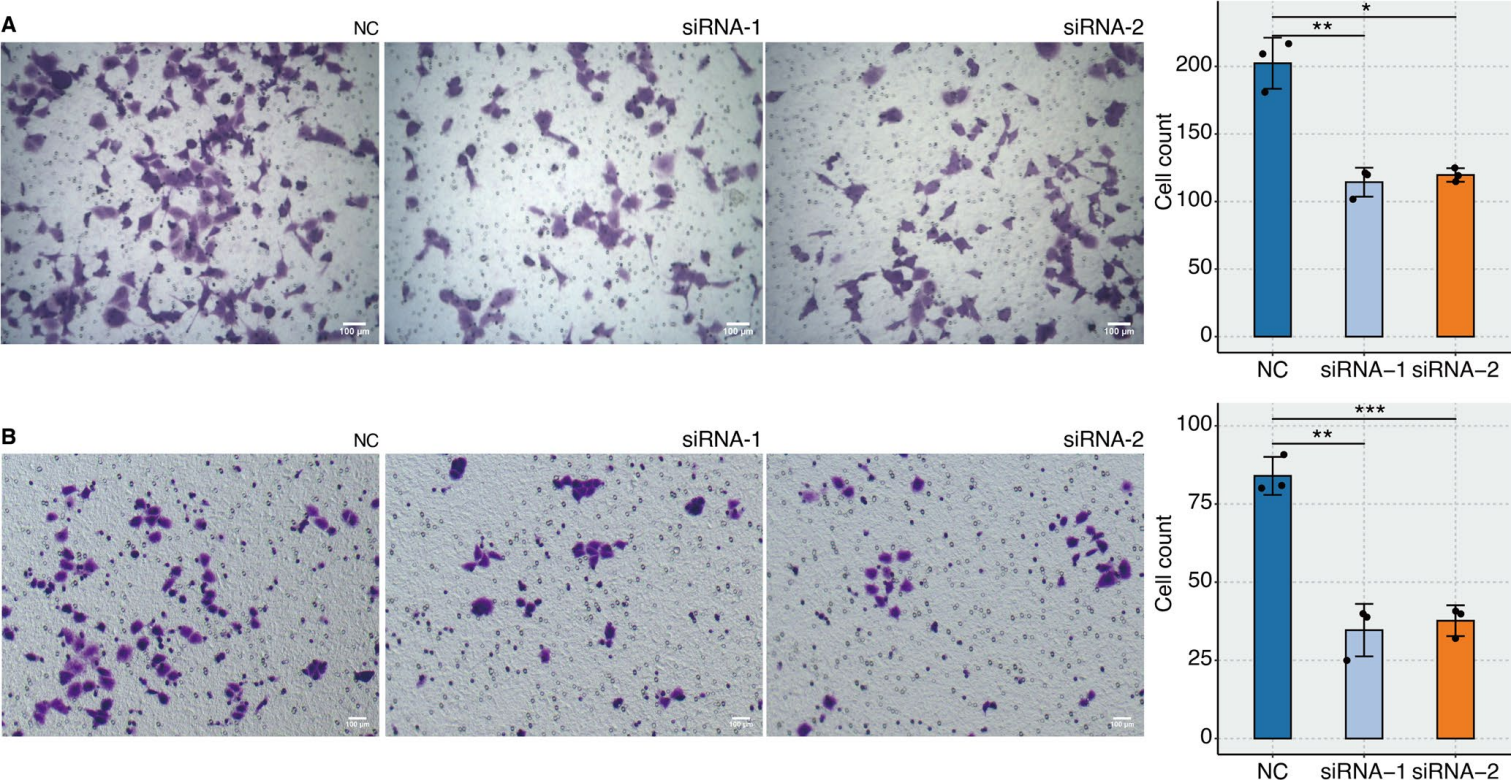
In vitro validation on TPD52. (A) Transwell assay reveals the migrated MCF‐7 cells in NC and two siRNA groups. (B) Transwell assay reveals the migrated M0 macrophages after coculture with MCF‐7 cells in NC and two siRNA groups.

RT‐qPCR assay revealed that the relative RNA expression of TPD52 was significantly lower in the three siRNA groups than in the NC group (Figure 6A). The OD value of MCF‐7 cells was significantly lower in the two siRNA groups than in the NC group by CCK‐8 assay (Figure 6B). EdU assay revealed that the proliferated MCF‐7 cells were significantly lower in the two siRNA groups than in the NC group (Figure 6C,D). Transwell assay revealed that the migrated MCF‐7 cells were significantly lower in the two siRNA groups than in the NC group (Figure 7A). Transwell assay revealed that the migrated M0 macrophages after coculture with MCF‐7 cells were significantly lower in the two siRNA groups than in the NC group (Figure 7B).

## 3 Discussion

In this study, we explored the role of TPD52 as a critical immune regulator in BRCA using machine learning–based methodologies and scRNA‐seq. Our findings reveal that TPD52 is associated with immune regulation and has significant implications for tumour behaviour and patient prognosis.

BRCA is characterised by its heterogeneity, complicating treatment strategies and patient outcomes [7]. The TME, comprising various cell types and their interactions, plays a pivotal role in tumour progression and immune evasion. Our analysis demonstrated distinct immune escape (IE) patterns within the BRCA microenvironment, emphasising the necessity of understanding these interactions for improved therapeutic approaches. Clusters associated with poor immune infiltration or high levels of immune evasion may correlate with worse clinical outcomes, such as shorter survival times or reduced response to immunotherapies. Understanding these patterns can inform prognostic assessments and help guide treatment strategies. Clustering analysis facilitates the development of personalised treatment approaches. By tailoring therapies based on an individual's immune profile, clinicians can enhance the efficacy of treatments and reduce unnecessary side effects.

TPD52 (tumour protein D52) is a gene that encodes a protein implicated in various cellular processes, including tumorigenesis, cell proliferation and immune regulation [8]. Originally identified as a tumour‐associated antigen, TPD52 has drawn attention in cancer research due to its potential role in the development and progression of several malignancies, including BRCA [9]. In prostate cancer, chaperone‐mediated autophagy is modulated by acetylation‐dependent regulation of TPD52 [10]. By reducing TPD52 expression, the androgen receptor inhibits the invasion of lung cancer and boosts the cisplatin response [11]. Notably, TPD52 is a survival factor for breast cancer cells that ERBB2 has increased [9]. The identification of TPD52 as a hazardous marker is particularly noteworthy. Machine learning has been widely used in cancer research [12]. Both CoxBoost and RSF enhance the ability to predict patient outcomes by identifying key prognostic factors among a large set of variables [13]. In the manuscript, these methods are employed to analyse the significance of intersected genes, including TPD52, thus providing a more accurate assessment of survival probabilities and risk stratification for BRCA patients. Our comprehensive analysis, including univariate Cox regression and machine learning techniques, established a correlation between high TPD52 expression and reduced survival rates across multiple datasets (TCGA, METABRIC, GSE9893). This finding aligns with emerging data suggesting that TPD52 may influence immune cell infiltration and modulate the tumour immune landscape, potentially exacerbating tumour aggressiveness.

Functional annotation through GSEA highlighted several oncogenic and immunogenic pathways associated with TPD52. Notably, pathways such as the PD‐1/PD‐L1 checkpoint [14] and TGF‐beta signalling [15] are critical in mediating immune responses and have been implicated in cancer progression. These results suggest that TPD52 may serve as a regulatory node within these pathways, warranting further investigation for its potential as a therapeutic target.

Additionally, our exploration of mutation characteristics related to TPD52 revealed significant mutation patterns in BRCA patients with high TPD52 expression. The association of TPD52 with specific mutation sites and high mutation rates may indicate its role in driving tumour evolution and heterogeneity. This underscores the importance of integrating genetic profiling with immune landscape analysis in understanding tumour dynamics [16].

The immune characteristics associated with TPD52 were also compelling. We observed a decreased abundance of various immune cell types in patients with elevated TPD52 levels, alongside negative correlations with immune and stromal scores. This suggests that TPD52 may contribute to an immunosuppressive TME, potentially aiding in immune escape and tumour progression [17].

The in vitro validation of TPD52's role further solidified our findings. Using siRNA to downregulate TPD52 expression in MCF‐7 cells resulted in notable cell proliferation and migration reductions, supporting the hypothesis that TPD52 is integral to tumour aggressiveness. Moreover, the effect of TPD52 on macrophage migration after coculture with MCF‐7 cells indicates its influence on immune cell dynamics within the TME.

In conclusion, our study provides novel insights into the role of TPD52 as an immune regulator in BRCA. TPD52 represents a promising target for therapeutic intervention and a valuable biomarker in breast cancer. Its ability to influence immune responses and correlate with patient prognosis underscores its potential utility in enhancing treatment strategies. Continued research into TPD52's mechanisms and applications will be essential for integrating this knowledge into clinical practice, ultimately improving outcomes for breast cancer patients. Future research should focus on elucidating the precise mechanisms through which TPD52 influences immune responses and tumour behaviour, which could pave the way for innovative therapeutic strategies in BRCA management.

## 4 Materials and Methods

### 4.1 In Silico Analysis

The Cancer Genome Atlas (TCGA), Molecular Taxonomy of Breast Cancer International Consortium (METABRIC) and GSE9893 [18] datasets were collected. We retrieved the Fragments Per Kilobase of transcript per Million mapped reads (FPKM)‐normalised values from the TCGA dataset and converted them into TPM format, making the data more comparable between samples and like microarray data. The GSE9893 dataset was retrieved from the Gene Expression Omnibus (GEO) database. The METABRIC dataset was retrieved from the cBioPortal database. The single‐cell sequencing (scRNA‐seq) dataset, GSE176078, was retrieved from the GEO database. Single‐cell analyses were performed using the R package Seurat, and the cutoff for single‐cell difference analysis was an absolute value of log fold change greater than 0.25 and a corrected p less than 0.05. Immune escape (IE)‐related genes were collected [19]. The R package Seurat was used to process the scRNA‐seq dataset [20]. Univariate Cox regression analysis was performed to identify the prognostic IE‐related genes. The R package ConsensusClusterPlus was used to identify the IE patterns using the k‐means method. Machine learning CoxBoost and random survival forest (RSF) [21] were used to reduce intersected genes' dimension. CoxBoost is a machine learning algorithm specifically designed for survival analysis. It enhances the traditional Cox proportional hazards model by incorporating boosting techniques, allowing for the identification of key prognostic factors among a large set of variables. RSF is an ensemble learning method that adapts the random forest algorithm for survival data. It constructs multiple decision trees and aggregates their predictions, making it robust against overfitting and capable of handling complex interactions among variables. The R package pRRophetic predicted chemotherapy drug responses based on the Genomics of Drug Sensitivity in Cancer (GDSC) [22]. GISTIC 2.0 analysis was performed on TPD52 [23]. The R package maftools was used to generate the mutation landscape related to TPD52 [24]. The R package survminer was used to create the survival curves of IE patterns and TPD52‐based groups. Gene set enrichment analysis (GSEA) was performed on TPD52 [25, 26]. The R package estimate was used to calculate the immune score, stromal score and Estimation of Stromal and Immune cells in Malignant Tumours using Expression (ESTIMATE) score [27]. The R package ComplexHeatmap was used to create a heatmap of the immune modulators related to TPD52 [28]. The TIMER algorithm was used to calculate the abundance of immune cells [29].

### 4.2 In Vitro Validation

iCell provided the cell lines MCF‐7 and THP‐1. They were grown in DMEM and 1640 media respectively. THP‐1 cells were polarised into M0 macrophages after exposure to 320 nM of phorbol 12‐myristate 13‐acetate (PMA) for 6 h at 37°C. siRNA sequences of TPD52 are as follows: human‐TPD52‐siRNA1:GCATGAACCTGATGAATGAAT; human‐TPD52‐siRNA2:GCAGAGTTAGTTCAGCTAGAA; human‐TPD52‐siRNA3:GAAGAGCTAAGAAGAGAACTT. RT‐qPCR assay was conducted to detect the relative RNA expression of TPD52. CCK‐8 and EdU assays were conducted to explore the proliferation ability of MCF‐7. Transwell assay was conducted to explore the migration of the ability of MCF‐7. After coculture with MCF‐7, Transwell assay was conducted to explore the migration of the ability of M0 macrophages.

### 4.3 Statistical Analysis

R was used for all statistical analyses. The Wilcoxon test was employed for data that was not regularly distributed, and the Student's t‐test was used to compare normally distributed variables between the two groups. P‐values below 0.05 were regarded as statistically significant.

## Author Contributions

X.Z. conceived, designed and supervised the study. J.X. and X.Z. performed the data analysis. J.X. and X.Z. wrote the manuscript. J.X. and X.Z. conducted the experiments. All authors read and approved the final manuscript.

## Ethics Statement

Review and/or approval by an ethics committee was not needed for this study because no patient or animal experiment was involved. Informed consent was not required for this study because no participants or patients were involved.

## Consent

The authors have nothing to report.

## Conflicts of Interest

The authors declare no conflicts of interest.

## Supporting information


**Data S1**.

## Data Availability

All data generated and/or analyzed during this study are available from the corresponding author upon reasonable request.
