# Supplementary figures and images for: TPD52 as a Therapeutic Target Identified by Machine Learning Shapes the Immune Microenvironment in Breast Cancer
Source: J Cell Mol Med. 2025 Jan 5;29(1):e70333. doi: 10.1111/jcmm.70333 (PMC11702390; doi:10.1111/jcmm.70333)

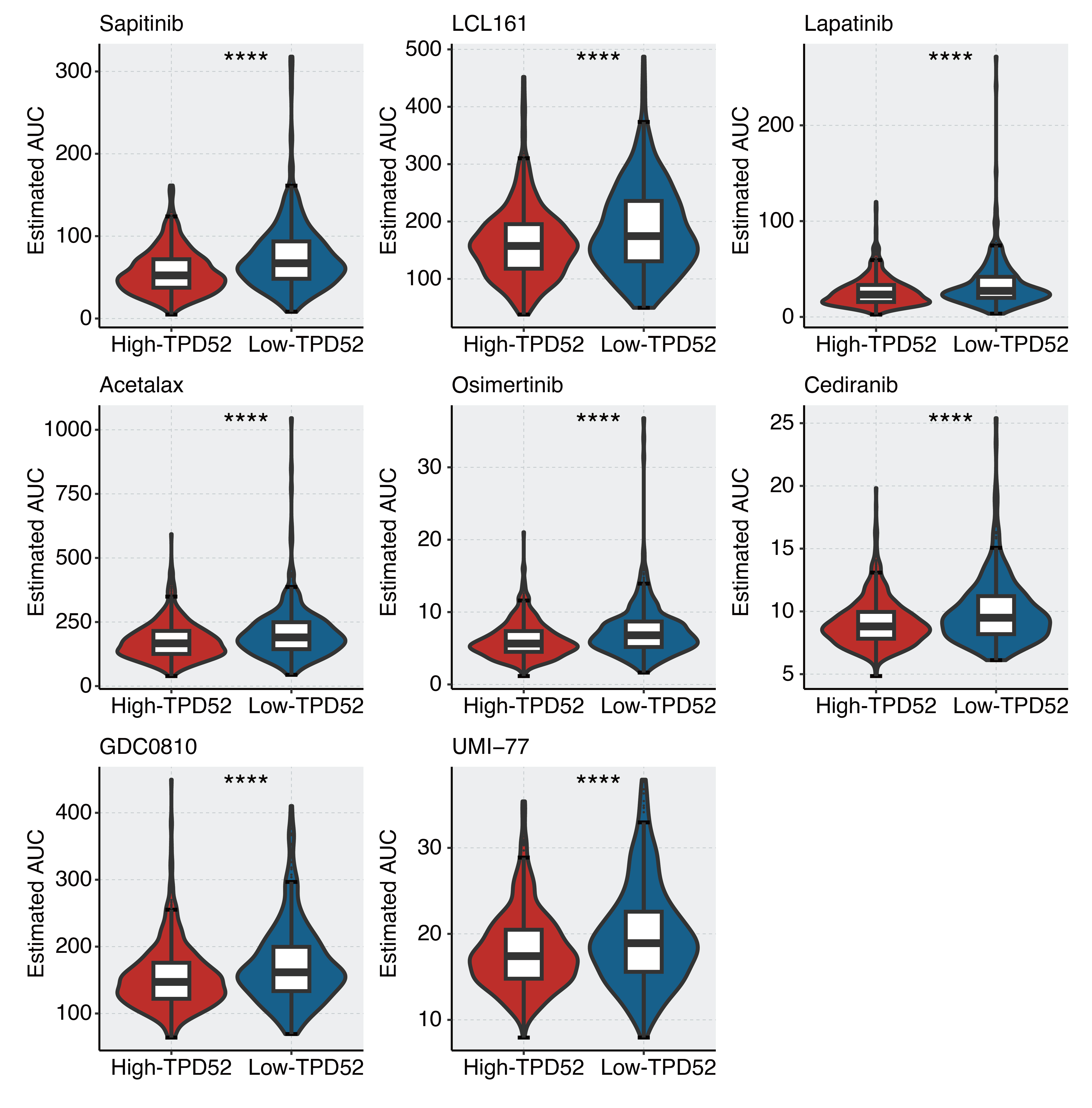


**Figure S1. The drug responses were predicted based on TPD52.**

Supplement: Supplementary file 1 — Data S1. [file JCMM-29-e70333-s001.docx]
